# New Eco-Friendly and Low-Energy Synthesis to Produce ZnO Nanoparticles for Real-World Scale Applications

**DOI:** 10.3390/nano13172458

**Published:** 2023-08-30

**Authors:** Giuliana Taglieri, Valeria Daniele, Valentina Maurizio, Gabriel Merlin, Cristina Siligardi, Marie Capron, Claudia Mondelli

**Affiliations:** 1 Department of Industrial and Information Engineering and Economics, University of L’Aquila, Piazzale E. Pontieri 1, Monteluco di Roio, Roio Poggio, 67100 L’Aquila, AQ, Italy; valemau99@gmail.com; 2 Department of Chemical Sciences, University of Padova, Via Marzolo 1, 35131 Padova, PD, Italy; gabriel.merlin@phd.unipd.it; 3 Department of Engineering “Enzo Ferrari”, University of Modena and Reggio Emilia, Via P. Vivarelli 10, 41125 Modena, MO, Italy; cristina.siligardi@unimore.it; 4 ESRF, 71 Avenue des Martyrs, 38042 Grenoble, CEDEX 9, France; marie.capron@esrf.fr; 5 Paternship for Soft Condensed Matter PSCM, 71 Avenue des Martyrs, 38042 Grenoble, CEDEX 9, France; 6 CNR-IOM-OGG, Institut Laue Langevin, 71 Avenue des Martyrs, 38042 Grenoble, CEDEX 9, France; mondelli@ill.fr

**Keywords:** ion exchange process, scalable synthesis, zinc oxide nanoparticles, simonkolleite, XRD, HRTEM, FESEM, AFM, BET

## Abstract

This paper presents an original and sustainable method for producing ZnO nanoparticles (NPs) in response to global challenges (low energy requirements, low environmental impact, short production times, and high production yield). The method is based on an ion exchange process between an anionic resin and an aqueous ZnCl_2_ solution; it operates in one step at room temperature/ambient pressure without the need for complex apparatus or purification steps. From the kinetics, we observed the formation of pure simonkolleite, a zinc-layered hydroxide salt (Zn_5_(OH)_8_Cl_2_·H_2_O), after only 5 min of reaction. This compound, used elsewhere as a ZnO precursor after calcination at high temperatures, here decomposes at room temperature into ZnO, allowing extraordinary savings of time and energy. Finally, in only 90 min, pure and crystalline ZnO NPs are obtained, with a production yield > 99%. Several types of aggregates resulting from the self-assembly of small hexagonal platelets (solid or hollow in shape) were observed. Using our revolutionary method, we produced almost 10 kg of ZnO NPs per week without any toxic waste, significantly reducing energy consumption; this method allows transferring the use of these unique NPs from the laboratory environment to the real world.

## 1. Introduction

Zinc oxide (ZnO) is a unique semiconductor material with widespread uses, all related to its peculiar properties, which include high chemical stability, a high electrochemical coupling coefficient, a broad radiation absorption range, high photostability, a low environmental impact related to biodegradability, low toxicity, and biocompatibility [1]. As a result, there has been a growing technological and economic interest in ZnO, fostering a plethora of uses in different areas, including in the rubber industry as a vulcanization activator [2,3,4], for UV absorption [5,6,7], and as a metal surface treatment [8]; in optoelectronics and laser technology [9] as a sensor and energy generator in hydrogen production [10]; and in biomedical applications [11,12]. The possibility of synthesizing nanostructured ZnO has been enhanced by the introduction of nanotechnology, which facilitates the production of zinc oxide nanoparticles (ZnO NPs) by simply changing the synthetic parameters, such as temperature, pH, or solvent [11]. The obtained ZnO NPs had different morphologies and crystal sizes and appeared to be characterized by unique and versatile properties, making them more competitive candidates than their corresponding bulk materials. ZnO NPs’ potential uses are now promising in various industrial, health, chemical, and consumer cosmetics fields. For example, ZnO NPs could be used as sunscreen agents due to their excellent ultraviolet (UV) absorbing properties and transparency to visible light [13,14,15]; as photoluminescence agents in biosensors [16,17]; as field-emission devices [18] in catalysis and photocatalysis [19,20]; as antibacterial and anticancer agents due to their ability to induce ROS generation [21,22,23]; and as singular drug carrier systems and medical filling materials due to their relative biocompatibility and reduced toxicity compared with other metal oxide NPs [13,16,23,24,25].

As reported in the literature, there are two approaches to nanoparticle synthesis: top-down and bottom-up. The top-down approach is based on the milling process of large macroscopic particles, reducing them to a nanoscale level through plastic deformation [26]. This technique has several limitations in terms of large-scale NP production, including a long processing time and high cost [27]. The bottom-up approach is based on physical and chemical methods, such as sol–gel and hydrothermal methods [28,29], chemical vapor deposition [30], microemulsion techniques [31], and laser ablation [32]. These methods, although allowing ZnO NP synthesis with different morphologies, often require expensive equipment, high pressure/temperature, capping and stabilizing agents, and toxic chemical reagents, which are harmful both to humans and the environment [27,33].

Considering the ever-growing attention towards environmentally friendly processes [34,35], in the last few years, researchers have developed methods to produce metal oxide NPs using greener and more cost-effective technologies based on biological methods [27,33,36,37,38,39,40]. However, some of these processes, although promising and less hazardous than chemical and physical methods, pose concerns regarding the large-scale production of ZnO NPs in relation to the complexity of the biological extracts acting as a barrier to the elucidation of the formation reactions/mechanism that occur during synthesis processes [41].

Paying attention to key environmental and sustainable credentials and widespread application needs, in this paper, we propose an original, eco-friendly, time- and energy-saving method to produce pure ZnO NPs with a high yield using a scalable procedure. This method is based on an ion exchange process already patented to produce different metal oxide/hydroxide NPs [42,43] that occurs in water, at room temperature and ambient pressure, and between a zinc chloride aqueous solution and an anionic exchange resin (OH^−^ form). The ion exchange between the chlorides in solution and the hydroxyl ions, initially present on the resin surface, exhibits extremely fast kinetics that lead, in supersaturated conditions, to a burst nucleation of a zinc-layered hydroxide compound, simonkolleite, which decomposes during synthesis, completely transforming at room temperature into pure and small ZnO NPs. The exchange process stops when the resin is exhausted. Both the fast formation of simonkolleite in one step and its decomposition into ZnO NPs at room temperature represent extraordinary results in terms of the efficacy and sustainability of this synthesis process, leading to a significant reduction in energy, time, and waste. In addition, the final NP suspension is separated from the resin, which, in turn, can be completely regenerated and reused for a new synthesis, facilitating a cyclic procedure.

The obtained ZnO NPs’ phase identification, structure, and crystallinity were investigated using several techniques, including X-ray powder diffraction (XRD), field emission scanning electron microscopy (FESEM), high-resolution transmission electron microscopy (HRTEM), and atomic force microscopy (AFM). Surface area (BET) measurements were also performed.

## 2. Materials and Methods

### 2.1. Materials

Zinc chloride (ZnCl_2_), with a purity > 98%, was supplied by Sigma Aldrich; the ion exchange resin Dowex Monosphere 550A was supplied by Sigma Aldrich (St. Louis, MO, USA) as translucent spherical beads having a particle size equal to 590 ± 50 μm. Sodium hydroxide (NaOH) pellets, with a purity > 98%, were supplied by Sigma Aldrich.

### 2.2. ZnO Nanoparticles Synthesis

ZnO nanoparticles were synthesized using an ion exchange process [42,43] in which a colourless aqueous ZnCl_2_ solution (1 M) was maintained in contact for 90 min under moderate stirring, with a proper amount of anionic resin in OH^−^ form in relation to its exchange capacity, 1.1 eq/L. During this time, Cl^−^ anions present in the aqueous solution were exchanged with the OH^−^ on the resin; in particular, Cl^−^ in the water was transferred to the resin, and OH- on the resin was exchanged into the water. Soon after the reaction began, a white precipitated phase appeared. After 90 min, we stopped the reaction and separated the resin from the produced aqueous suspension using a metallic sieve (180 μm aperture). Next, we placed the exhausted resin in a column, and an 8% wt NaOH aqueous solution was applied at a flow rate of approximately 1 L/min until the resin was completely regenerated and ready for use in a new synthesis cycle [44].

The kinetics of the ion exchange process were monitored over time; we measured the chloride ion concentration (*CC*) in the whole batch at different times (*t*) by taking samples at regular time intervals from the beginning to the end of the reaction (t = 0, 5, 15, 30, 45, 60, and 90 min). We measured *CC* values using an ion-sensitive electrode for Cl^−^ ions (Metrohm, Herisau, Switzerland). From these values, we obtained the ion exchange process yield (*Y*) at different synthesis times and the exchange rate (*R*) in time interval ∆*t*, according to the following formulas:

(1) $$Y\left(t\right)=\left(\frac{{CC}_{0}-CC(t)}{C_{0}}\right)*100$$

(2) $$R\left(t\right)=\left(\frac{CC(tx)-CC(ty)}{∆t}\right)$$

where *CC*_0_ is the chloride concentration at time *t* = 0, and *CC*(*tx*) and *CC*(*ty*) represent chloride ion concentration values at two consecutive times.

### 2.3. Characterization of the Produced Zinc Oxide Nanoparticles

We investigated the phase composition, structure, and crystallinity at different times during the exchange process using the X-ray diffraction (XRD) technique. XRD scans were recorded at room temperature using a X’Pert PRO diffractometer (PANalytical, Almelo, The Netherlands) with Cu-Kα radiation. We deposited 0.12 mL of the aqueous suspension samples on a zero-background sample holder and left them to dry at laboratory conditions (T = 20 °C; RH = 45%). XRD patterns were recorded in the 5–70° 2θ range, using a step size of 0.026° 2θ and a step time of 200 s. Each experimental diffraction pattern was elaborated using Profile Fit Software (High Score Plus software package, version 4.9, PANalytical, Almelo, The Netherlands); crystalline phases were attributed using the international ICDD and ICSD reference databases. X-ray data were fitted using the pseudo-Voight profile function and refined using Rietveld refinements [45]. To evaluate the average crystallite size, D_hkl_ of the ZnO crystals, XRD peak broadening analysis was carried out using the Debye–Scherrer formula [45,46].

The morphologies and dimensions of the produced NPs and their aggregates were investigated using a field emission scanning electron microscope (Gemini FESEM 500, ZEISS, Oberkochen, Germany) and a high-resolution transmission electron microscope with a 200 keV acceleration voltage (TEMFEG, Talos F200S G2, ThermoFisher, Boston, MA, USA). Samples were prepared by dropping aqueous suspension samples onto suitable SEM stabs or TEM grids. Suspension concentrations of 1 g/L and 0, V. 4.92 g/L were considered.

Atomic force microscopy (AFM) (Cypher, Asylum Research, available at the PSCM AFM platform in tapping mode) measurements were taken using a Cypher Asylum Research instrument using a cantilever Bruker model SNL 10 in tapping mode; some drops of the diluted suspension were dispersed under a nitrogen atmosphere on the flat surfaces of a mica substrate. In particular, mica discs must be cleaved to produce a clean surface before use as a substrate.

The Brunauer–Emmett–Teller (BET) surface area was determined using nitrogen adsorption measurements performed at 77 K with a ramp rate of 10 K/min using a Micromeritics ASAP2020 Plus system (Micromeritics, Norcross, GA, USA). The pore size distribution was determined using the isotherms’ desorption branch using the Barett–Joyner–Halenda (BJH) method.

## 3. Results and Discussion

**Figure 1:**
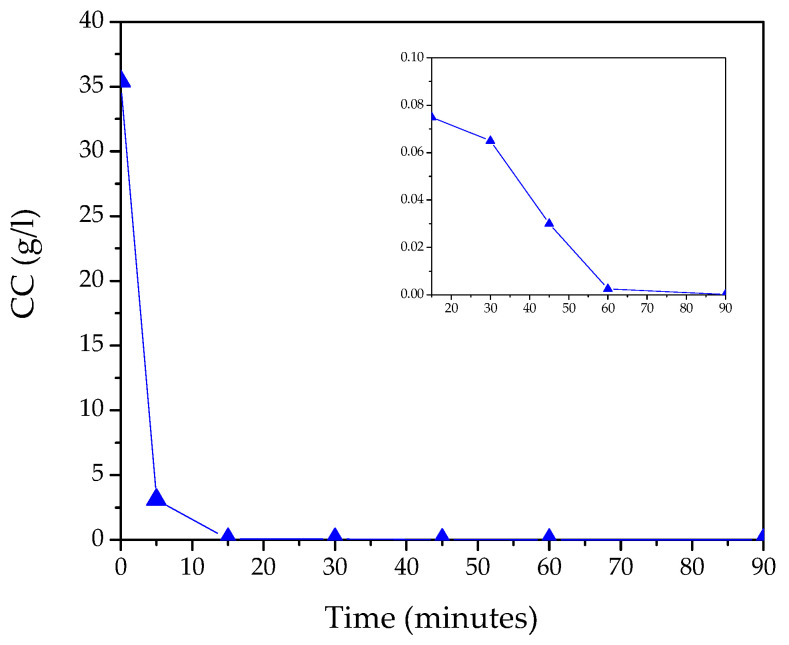
Kinetics of the ion exchange process in terms of the chloride concentration (*CC*) versus time. The inset shows the lowest values reported.

*CC* value measurements, which provide information about the kinetics of the ion exchange process, are shown in Figure 1.

Table 1 reports each single measurement in relation to the corresponding yield (*Y*) and exchange rate (*R*) values.

From these measurements, we observed a fast kinetics, leading in a few minutes to an almost complete reduction in CC values in solution; after only 5 min, we measured a yield, Y, of 91.2%, as shown in Table 1. At the end of the synthesis (after 90 min), we measured a residual CC value of 0.1 mg/L, denoting a complete transformation of the reagent and a production yield, Y, of 100%. From the CC values, we also observed a relevant difference in the exchange rates, R, underlying three different stages of kinetics: (1) from 0 to 5 min, we observed the highest rate R, equal to 6.5 g/L·min; (2) from 5 to 15 min, the R-value quickly decreased to 0.2 g/L·min; and (3) from 15 to 90 min, we found a slow rate of four orders of magnitude lower, which represented the reaction’s saturation value.

**Figure 2:**
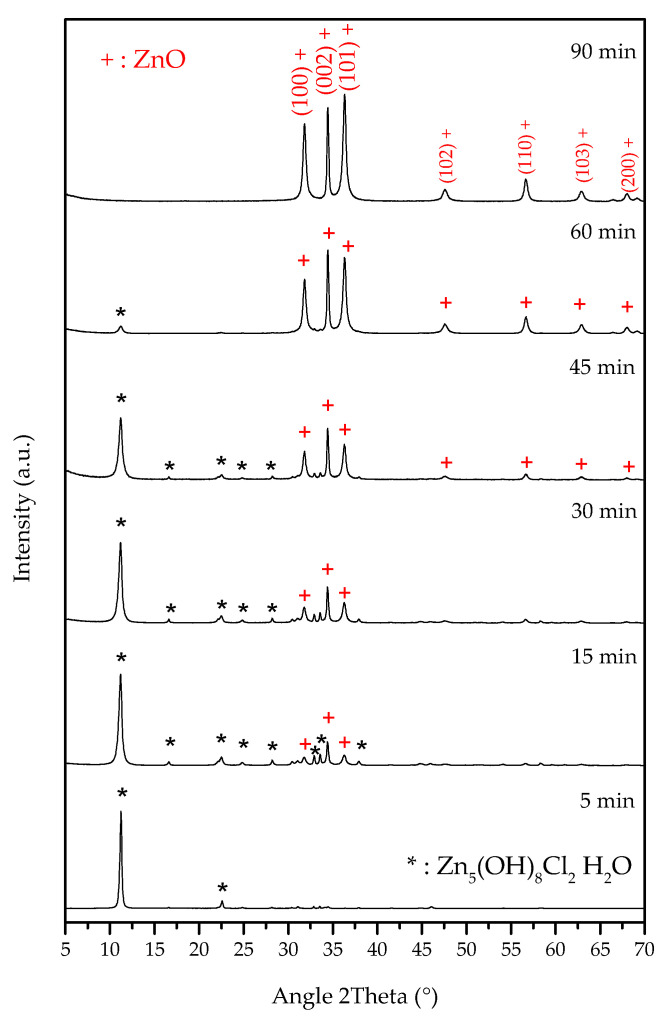
XRD patterns of the produced suspension at different times during the reaction of the ion exchange process. Legend: * Simonkolleite (Zn_5_ (OH)_8_ Cl_2_·H_2_O); ^+^ Zinc Oxide (ZnO).

XRD analyses performed on samples taken at different times during the reaction provided useful information about the phases formed during the ion exchange reaction. The XRD patterns shown in Figure 2 illustrate that, only 5 min after the reaction began, a crystalline phase formed, which was ascribed to pure simonkolleite, a zinc chloride hydroxide hydrate [47] (chemical formula: Zn_5_ Cl_2_ (OH)_8_·H_2_O (ICSD #98-009-5365)). No other phases were observed in the XRD spectra, neither crystalline nor amorphous. However, XRD analysis results showed that simonkolleite was not stable over time; 15 min after the reaction began, we observed a reduction in simonkolleite, together with a simultaneous formation of ZnO with a hexagonal wurtzite-type structure (ICSD# 98-005-7450). As the synthesis time increased, the ZnO amount increased, and the simonkolleite amount decreased. After 90 min, no other characteristic peaks or amorphous contribution were present other than the ZnO phase. Moreover, the XRD peaks were relatively broad, denoting a small average crystallite size of 20 nm, as calculated using the Debye–Scherrer equation. When the experimental relative intensities were compared with those of the ICSD reference pattern, we observed a preferential orientation of NPs along the (002) plane, as also evidenced in Figure 2. In particular, from 15 to 60 min, after synthesis began, the (002) lattice plane was predominant, underlining that the NP formation proceeded along this plane, giving rise to lamellar-shaped ZnO NPs [48]. This result was confirmed by the fact that the highest D_hkl_ value referred to the (002) plane, corresponding to 48.9 nm.

**Figure 3:**
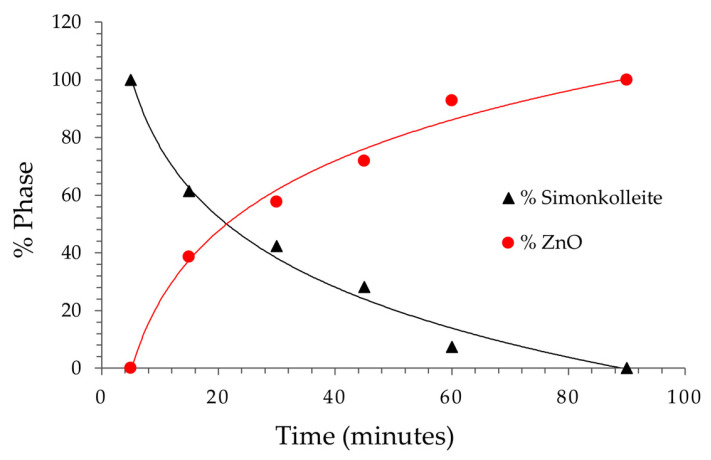
Graphical behaviour of the formed crystalline phases versus time.

Quantitative analysis results for the formed crystalline phases versus time, carried out using Rietveld refinements, are reported in Table 1 and graphically shown in Figure 3. These results, particularly as observed in graphical mode, are remarkable. In fact, interpolating the experimental data provided the fundamental information that the simonkolleite logarithmic decrease (R^2^ = 0.9883) was directly related to the symmetric ZnO increase (R^2^ = 0.9883), according to the following equations:

S (%) = −35.05 ln(t) + 157.39

ZnO (%) = 35.05 ln(t) − 57.39

These results, together with *CC* values reported in Table 1, are evidence of the novelty and originality of this synthesis, reported for the first time here. In principle, we expected that the fast decrease in chlorides, which occurred at the beginning of the exchange reaction, corresponded to their absorption on the surface of the anionic exchange resin [44,49,50,51,52], as described by the following formula:

ZnCl_2_ + 2R(OH^−^) → 2R(Cl^−^) + Zn(OH)_2_ (3)

Nevertheless, the XRD results evidenced that simonkolleite formation was primarily responsible for the subtraction of chlorides from the solution. As reported in the previous literature [53], the high concentration of Zn^2+^ and Cl^−^ ions, together with the hydroxyls present at the starting steps of the reaction, favoured the formation of simonkolleite according to the following chemical reaction:

5Zn^2+^ + 8OH^−^ + 2Cl^−^ + H_2_O → Zn_5_(OH)_8_Cl_2_·H_2_O (4)

However, as the exchange reaction progressed, excess hydroxyl ions in solution allowed for an alkaline ambient, which favoured the presence of zincate ions, such as Zn(OH)_4_^2−^, acting as precursors for the nucleation and crystal growth of ZnO [47,54,55] according to the following reaction:

Zn(OH)_4_^2−^ → ZnO + H_2_O + 2OH^−^ (5)

In summary, XRD analysis underlined the direct formation of ZnO from simonkolleite at room temperature. So, although simonkolleite is a crystalline solid insoluble in water that is used as a precursor for ZnO formation only after calcination at high temperatures [56,57], the synthesis method here proposed exhibits, in an innovative way, that ZnO originates from simonkolleite at ambient temperature, with noteworthy savings of time and energy. This extraordinary result, never observed before, could be due to the resin favouring a continuous alkaline ambient (see Formula (5)) that could exert a driving force in simonkolleite’s gradual dissolution.

The ZnO NPs obtained 90 min after the synthesis process began were characterized from a morphological and dimensional point of view using several microscopic investigations, including FESEM, TEM/HRTEM, and AFM techniques.

**Figure 4:**
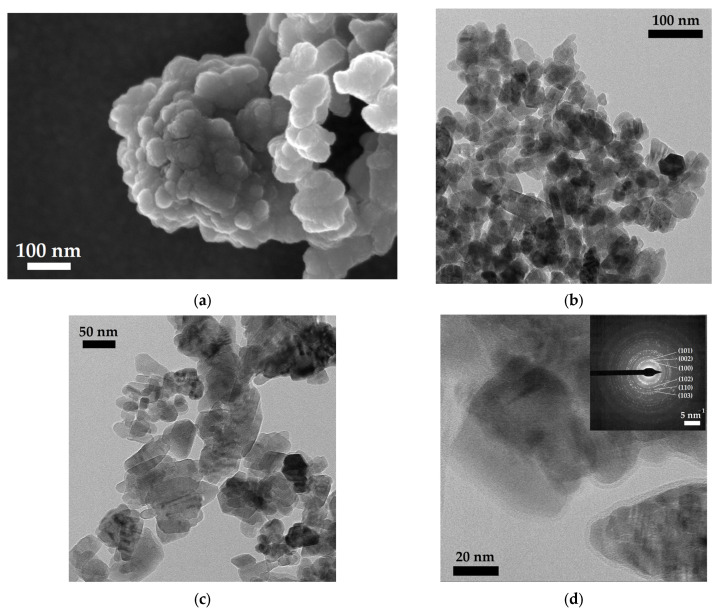
(**a**) FESEM image of ZnO nanoparticle agglomerates. (**b**,**c**) HRTEM images showing ZnO NPs generally less than 50 nm in size, characterized by different morphologies (from hexagonal to triangular) and distributed in elongated agglomerates. (**d**) At higher magnification, a particle of approximately 50 nm resulted in a self-assembly of primary ZnO NPs, each exhibiting the typical ZnO diffraction pattern (see the SAED image in the inset).

FESEM images of the as-synthesized ZnO NPs (Figure 4) show the presence of several agglomerates composed of 30–40 nm hexagonal or pseudo-hexagonal clusters, arranged in turn by a dense aggregation of small ZnO NPs approximately 10 nm in size.

HRTEM images provided a more detailed observation of some morphologies, as can be seen in Figure 4b. Well-defined hexagonal lamellar morphologies are visible, as are triangular-shaped NPs and several elongated agglomerates. The previous literature indicates that both triangular and elongated morphologies can be recognized when a preferential orientation effect along the (002) plane is present [58,59], as also observed in our XRD analyses. In fact, as the (002) surfaces were polar surfaces in the Wurtzite crystal structure, the particles had a dipole moment along the *c*-axis, leading to a pile orientation along the (002) plane. In addition, as reported in the literature [60], the formation of agglomerates can be attributable to the high surface energy of ZnO NPs, especially when the synthesis is carried out in an aqueous medium. The presence of lamellar particles, tending to align along the basal plane, is better described by Figure 4c, in which we observed many 10 nm hexagonal NPs. Elongated agglomerates, as visible in Figure 4c, presented darker areas along the main axis, but not in a compact way, as if to signify the stacking of hollow lamellar blocks. This hypothesis is supported by the presence, in the same image, of hexagonal platelets of approximately 50 nm in size having a marked outer order and visibly piled on top of each other (see red arrows). Interestingly, the highly magnified image in Figure 4d shows the self-assembling of 10 nm-sized primary NPs exhibiting the typical ZnO diffraction pattern, as seen in the selected area electron diffraction (SAED) pattern in the inset.

**Figure 5:**
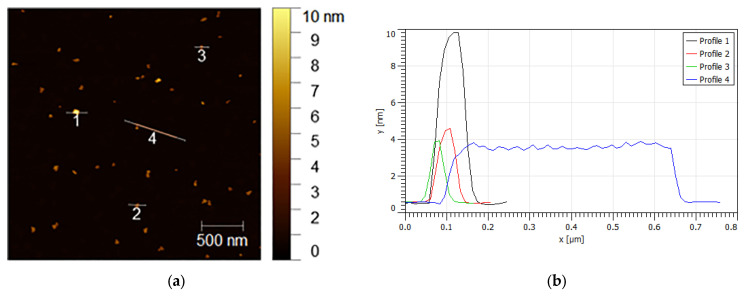

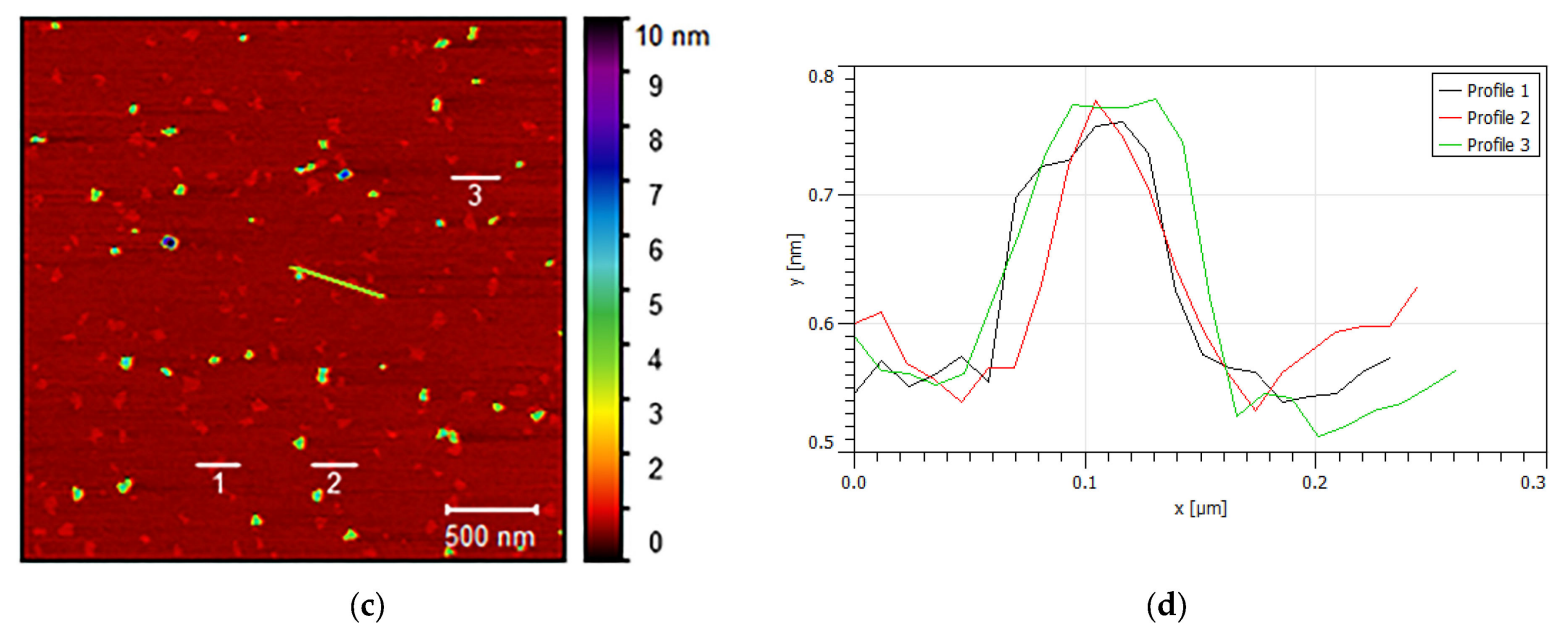
(**a**–**d**) AFM images of the ZnO nanoparticles, together with the corresponding profile analyses along the *Y*-axis, showing three thicknesses equal to approximately 10 nm, 4 nm, and 0.5 nm. (**b**) A clear rod-like morphology 600 nm long and only 4 nm thick, corresponding to profile 4, was also observed.

**Figure 6:**
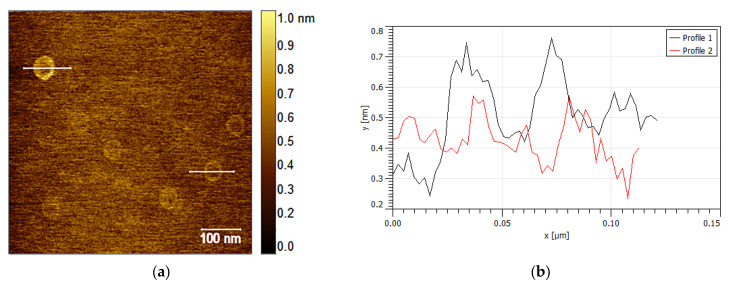

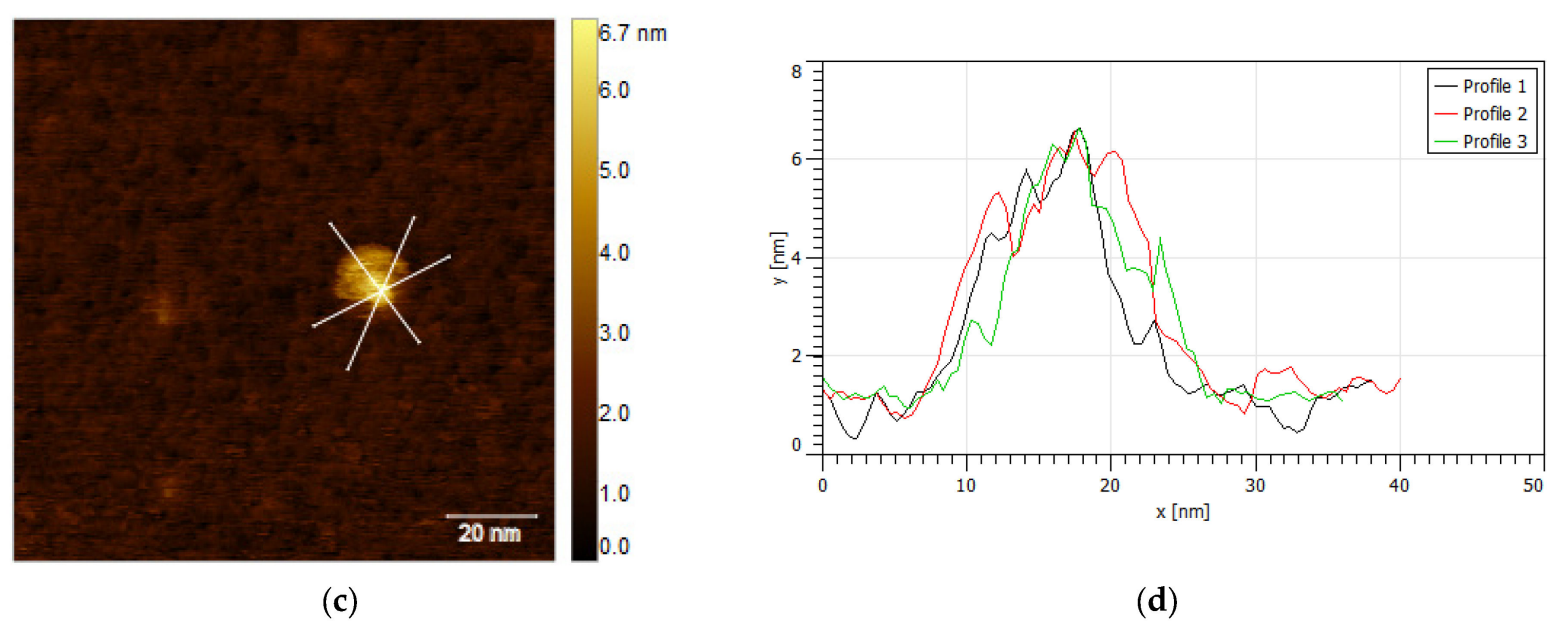
(**a**,**b**) AFM images, performed at higher magnification, of ZnO hexagonal hollow platelets of approximately 50 nm in size and 0.50 nm thick; (**c**,**d**) a conic-shaped morphology was also identified.

ZnO NPs’ thickness was analyzed using AFM at different magnifications, as shown in Figure 5 and Figure 6. At low magnification (Figure 5a–c), we observed that ZnO NPs possessed three thicknesses (approximately 10 nm, 4 nm, and 0.5 nm), as measured using a profile analysis along the *Y*-axis and emphasized by the colour gradation elaboration image (Figure 5b–d). In addition, a clear rod-like morphology, 600 nm long and only 4 nm thick, was observed (Figure 5b). However, from the profile analysis along the *Y*-axis, we noted an oscillating profile that could be ascribed to a regular stack of single NPs piled on top of one another with an interparticle spacing of approximately 50 nm.

When observed at higher magnifications, AFM investigations provide additional crucial insights regarding ZnO NP morphologies, as shown in Figure 6. In Figure 6a, several hexagonal hollow platelets are clearly visible; these were approximately 50 nm in size and extremely thin (approximately 0.50 nm), as measured using the Y-profile reported in Figure 6b. The observation of this morphology confirmed the hypothesis (described above) concerning the presence of elongated hollow aggregates composed of stacks of hexagonal hollow blocks. The last interesting morphology arising from the AFM analysis is shown in Figure 6c, together with three radial profiles along the *Y*-axis (Figure 6d). This morphology, together with the symmetric behaviour of the profile analysis, facilitated the identification of a conic shape, probably due to the overlap of a hollow hexagon and a solid particle, leading to the triangular shapes observed using TEM.

**Figure 7:**
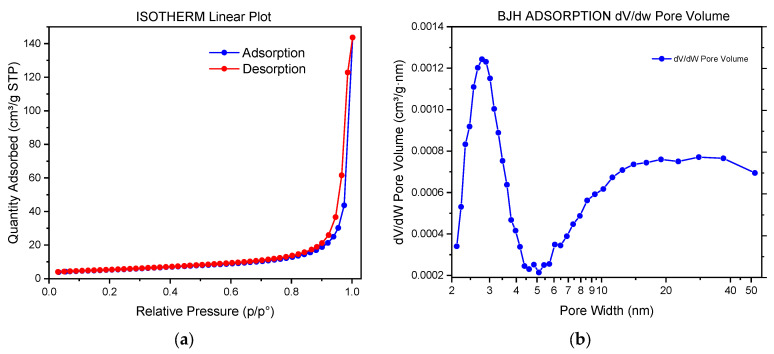
(**a**) Nitrogen adsorption–desorption isotherms for obtained ZnO dry powders; (**b**) Barrett–Joyner–Halenda (BJH) pore size distribution curve determined using the N2 desorption isotherm.

Surface area measurements for ZnO samples taken after 90 min are reported in Figure 7. Considering the total pore volume of the analyzed sample, it was clear that, according to the IUPAC classification, the adsorption isotherm was well matched to type IV, corresponding to the presence of micro- and meso-porosities (Figure 7a) [61]. Two cycles of hysteresis were noted, which suggested the presence of two different pore size distributions in different regions.

At higher relative pressure, 0.90–1.0 p/p°, the hysteresis can be related to an H3 hysteresis loop, attributable to slit-like pores or plate-like particles [62].

The pore size distribution, obtained using the BJH method and reported in Figure 7b, indicates that pores were primarily centred in the 2–50 nm range, confirming that the sample was composed of a mesoporous structure in agreement with the type IV adsorption isotherm [63]. The measured BET surface area was equal to 18.57 m^2^/g, and the data obtained were in agreement with the highest value typically reported in the literature [63,64,65,66,67,68,69,70].

However, the obtained BET results were mainly related to ZnO NP aggregates, without considering the contribution of primary NPs and sub-nanometric agglomerates, which were directly observed through HRTEM and AFM investigations.

## 4. Conclusions

The exceptional and multifunctional properties of zinc oxide nanoparticles (ZnO NPs) have increased interest in their application in numerous fields. However, the possibility of synthesizing ZnO NPs on a large scale will transfer our results to an industrial level and facilitate their extensive use as required in applicable fields. Simultaneously, the present global situation requires production procedures assuring low environmental impact, low energy use, and high production yields. Different approaches to producing ZnO NPs have been reported in the literature, but most require expensive equipment, high pressure and/or high temperature, toxic chemical reagents and/or organic substances, and long synthesis times; none of these factors are sustainable or eco-friendly.

In this paper, we present a revolutionary synthesis route that overcomes limitations related to the amount of ZnO NPs produced, as well as the sustainability of production processes and their impact on the environment. In particular, the extraordinarily fast kinetics of this innovative synthesis are fundamental to the high supersaturation conditions required for an immense nucleation with respect to growth, leading to the formation of nanostructured ZnO particles. Moreover, the cyclic procedure, together with the exchange process’ high yield (almost 10 kg NPs/week), represents the key to defining how to begin to scale the process to meet real-world requests.

Using different microscopes, we evidenced the presence of NP aggregates of different morphologies, from hexagonal lamellar platelets to triangular and hollow elongated structures, all composed of primary NPs ≤ 10 nm in size, with thicknesses ranging from 10 to 0.5 nm.

We are convinced that this method will have a significant impact on society and industry and inspire a new vision for the extensive industrial and medical applications of this impressive versatile material (ZnO NPs), fulfilling all market requests in terms of large quantities, sustainability, and a green approach.

## Figures and Tables

**Table 1:** Chloride concentrations (*CC*) at different times (*t*), corresponding production yields (*Y*), exchange rates (*R*) from the beginning to the end of synthesis, and quantitative analyses—elaborated using Rietveld refinements—of phases formed during the exchange reaction.

*t* (Minutes)	*CC* (g/L)	*Y* (%)	*R* (g/L·min)	% of Crystalline Phases Using Rietveld Analysis
0	35.4	-		
5	3.1	91.2	6.5	100% S
15	0.075	99.8	0.2	61.4% S–38.6% Z
30	0.065	99.8	0.0003	42.3% S–57.7% Z
45	0.030	99.9	0.0008	28.2% S–71.8% Z
60	0.0025	100.0	0.0005	7.3% S–92.7% Z
90	0.0001	100.0	0.00003	100% Z

Legend: S: simonkolleite; Z: ZnO.

## Data Availability

Not applicable.
